# Supplementary material for: Detailed molecular and epigenetic characterization of the pig IPEC-J2 and chicken SL-29 cell lines
Source: iScience. 2023 Feb 20;26(3):106252. doi: 10.1016/j.isci.2023.106252 (PMC10018572; doi:10.1016/j.isci.2023.106252)
Supplement: Data S1. Complete homer output for identified motifs in Pig IPECJ-2, related to Table 2 — Homer motif analysis results for histone modifications H3K4me1, H3K4me3, H3K27ac, and enhancer elements of pig IPECJ2 cell line. P-values >1e-10 are possible false positives. Within each folder (e.g. peak_files_CTCF) are the html files showing the identified motifs when using homer (e.g. homerResults.html). [file mmc2.zip › S5/Pig_IPECJ_2/peak_files_H3K4me3/homerResults/motif5.similar.html]

motif5

## Information for motif5

T
A
G
C
A
T
C
G
A
C
T
G
T
A
C
G
T
C
G
A
T
C
G
A
C
G
T
A
T
G
C
A
  
Reverse Opposite:  

A
C
G
T
G
C
A
T
A
G
C
T
A
G
C
T
A
T
G
C
T
G
A
C
T
A
G
C
A
T
C
G
  

|  |  |
| --- | --- |
| p-value: | 1e-154 |
| log p-value: | -3.561e+02 |
| Information Content per bp: | 1.437 |
| Number of Target Sequences with motif | 13924.0 |
| Percentage of Target Sequences with motif | 63.08% |
| Number of Background Sequences with motif | 14354.5 |
| Percentage of Background Sequences with motif | 54.25% |
| Average Position of motif in Targets | 151.4 +/- 85.6bp |
| Average Position of motif in Background | 149.6 +/- 101.0bp |
| Strand Bias (log2 ratio + to - strand density) | -0.0 |
| Multiplicity (# of sites on avg that occur together) | 1.38 |
| Motif File: | file (matrix) reverse opposite |

### Similar de novo motifs found

|  |  |  |  |  |  |  |  |
| --- | --- | --- | --- | --- | --- | --- | --- |
| Rank | Match Score | Redundant Motif | P-value | log P-value | % of Targets | % of Background | Motif file |
| 1 | 0.920 | T A C G T A G C T A C G T C A G T C A G C T G A T C G A C T G A T C G A T C G A | 1e-81 | -187.939458 | 37.32% | 31.23% | motif file (matrix) |
| 2 | 0.671 | A C T G G T C A G C T A A C G T G A C T A C G T A C G T C A G T A C G T A G T C G T A C G A T C A G T C | 1e-20 | -46.243694 | 0.14% | 0.02% | motif file (matrix) |
